# Oral delivery of nanomedicine for genetic kidney disease

**DOI:** 10.1093/pnasnexus/pgae187

**Published:** 2024-05-10

**Authors:** Yi Huang, Jonathan Wang, Valeria Mancino, Jessica Pham, Colette O’Grady, Hui Li, Kairui Jiang, Deborah Chin, Christopher Poon, Pei-Yin Ho, Georgina Gyarmati, János Peti-Peterdi, Kenneth R Hallows, Eun Ji Chung

**Affiliations:** Department of Biomedical Engineering, University of Southern California, Los Angeles, CA 90089, USA; Department of Biomedical Engineering, University of Southern California, Los Angeles, CA 90089, USA; Department of Medicine, Division of Nephrology and Hypertension, Keck School of Medicine, University of Southern California, Los Angeles, CA 90033, USA; USC/UKRO Kidney Research Center, Keck School of Medicine, University of Southern California, Los Angeles, CA 90033, USA; Department of Medicine, Division of Nephrology and Hypertension, Keck School of Medicine, University of Southern California, Los Angeles, CA 90033, USA; USC/UKRO Kidney Research Center, Keck School of Medicine, University of Southern California, Los Angeles, CA 90033, USA; Department of Biomedical Engineering, University of Southern California, Los Angeles, CA 90089, USA; Department of Medicine, Division of Nephrology and Hypertension, Keck School of Medicine, University of Southern California, Los Angeles, CA 90033, USA; USC/UKRO Kidney Research Center, Keck School of Medicine, University of Southern California, Los Angeles, CA 90033, USA; Department of Biomedical Engineering, University of Southern California, Los Angeles, CA 90089, USA; Department of Biomedical Engineering, University of Southern California, Los Angeles, CA 90089, USA; Department of Biomedical Engineering, University of Southern California, Los Angeles, CA 90089, USA; Department of Medicine, Division of Nephrology and Hypertension, Keck School of Medicine, University of Southern California, Los Angeles, CA 90033, USA; USC/UKRO Kidney Research Center, Keck School of Medicine, University of Southern California, Los Angeles, CA 90033, USA; Department of Physiology and Neuroscience, and Medicine, Zilkha Neurogenetic Institute, University of Southern California, Los Angeles, CA 90033, USA; Department of Physiology and Neuroscience, and Medicine, Zilkha Neurogenetic Institute, University of Southern California, Los Angeles, CA 90033, USA; Department of Medicine, Division of Nephrology and Hypertension, Keck School of Medicine, University of Southern California, Los Angeles, CA 90033, USA; USC/UKRO Kidney Research Center, Keck School of Medicine, University of Southern California, Los Angeles, CA 90033, USA; Department of Biomedical Engineering, University of Southern California, Los Angeles, CA 90089, USA; Department of Medicine, Division of Nephrology and Hypertension, Keck School of Medicine, University of Southern California, Los Angeles, CA 90033, USA; Department of Chemical Engineering and Materials Science, University of Southern California, Los Angeles, CA 90089, USA; Department of Surgery, Division of Vascular Surgery and Endovascular Therapy, Keck School of Medicine, University of Southern California, Los Angeles, CA 90033, USA; Department of Stem Cell Biology and Regenerative Medicine, University of Southern California, Los Angeles, CA 90089, USA; Norris Comprehensive Cancer Center, University of Southern California, Los Angeles, CA 90033, USA; Bridge Institute, University of Southern California, Los Angeles, CA 90089, USA

**Keywords:** kidney targeting, oral delivery, nanomedicine, drug delivery, chronic disease

## Abstract

Chronic and genetic kidney diseases such as autosomal dominant polycystic kidney disease (ADPKD) have few therapeutic options, and clinical trials testing small molecule drugs have been unfavorable due to low kidney bioavailability and adverse side effects. Although nanoparticles can be designed to deliver drugs directly to the diseased site, there are no kidney-targeted nanomedicines clinically available, and most FDA-approved nanoparticles are administered intravenously which is not ideal for chronic diseases. To meet these challenges of chronic diseases, we developed a biomaterials-based strategy using chitosan particles (CP) for oral delivery of therapeutic, kidney-targeting peptide amphiphile micelles (KMs). We hypothesized that encapsuling KMs into CP would enhance the bioavailability of KMs upon oral administration given the high stability of chitosan in acidic conditions and mucoadhesive properties enabling absorption within the intestines. To test this, we evaluated the mechanism of KM access to the kidneys via intravital imaging and investigated the KM biodistribution in a porcine model. Next, we loaded KMs carrying the ADPKD drug metformin into CP (KM-CP^-met^) and measured in vitro therapeutic effect. Upon oral administration in vivo, KM-CP^-met^ showed significantly greater bioavailability and accumulation in the kidneys as compared to KM only or free drug. As such, KM-CP^-met^ treatment in ADPKD mice (*Pkd1^fl/fl^*;*Pax8-rtTA*;*Tet-O-Cre* which develops the disease over 120 days and mimics the slow development of ADPKD) showed enhanced therapeutic efficacy without affecting safety despite repeated treatment. Herein, we demonstrate the potential of KM-CP as a nanomedicine strategy for oral delivery for the long-term treatment of chronic kidney diseases.

**Significance Statement**

Genetic kidney diseases such as autosomal dominant polycystic kidney disease (ADPKD) are lifelong diseases that have no nanomedicine options. Here, we report the development of nanotherapeutics based on kidney-targeting peptide amphiphile micelles that significantly improve drug bioavailability to the kidneys and can be taken orally, a critical requirement for chronic diseases such as ADPKD. This nanomedicine-based strategy is the first of its kind, offering a transformative, technological advancement for patients with chronic kidney disease.

## Introduction

Autosomal dominant polycystic kidney disease (ADPKD) is a chronic kidney disease and the most common inherited kidney disease, affecting 12.5 million people worldwide (1). The disease is caused by mutations in *PKD1* or *PKD2* and is characterized by overproliferation of renal cells and uncontrolled cyst growth that leads to a loss in kidney function and renal failure (2–5). Tolvaptan, the only FDA-approved drug for ADPKD which was approved in 2018, inhibits adenylyl cyclase and the cyclic AMP (cAMP) pathway and decreases fluid secretion and cell proliferation, thereby slowing ADPKD. However, ADPKD patients that take tolvaptan treatment can experience considerable side effects such as polyuria, thirst, nausea, and drug-induced liver damage leading to a patient dropout rate of 25% (6–12).

In addition to tolvaptan, other drug candidates have shown therapeutic promise in preclinical studies; however, similar to tolvaptan, enthusiasm was dampened during clinical trials due to severe side effects. For example, bardoxolone methyl, which restores mitochondrial function, inhibits inflammation, and showed benefits in reducing cystogenesis in ADPKD cells (13), induced cardiac and gastrointestinal disorders and led to early clinical trial discontinuation (14). Additionally, although metformin, an AMPK activator and mTOR inhibitor that is currently FDA approved for diabetes, was found to significantly inhibit disease progression in PKD mice (15), clinical trials testing metformin in ADPKD patients only slightly reduced the glomerular filtration rate decline (16) and caused hypoglycemia and lactic acidosis (17–19). Therefore, developing kidney-targeting drug delivery strategies has the potential to benefit diseases like ADPKD by directly transporting drugs to the disease site and simultaneously inhibiting off-target side effects.

**Scheme 1.**
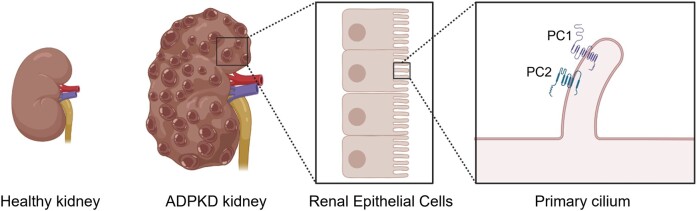
Schematic of oral delivery strategy of KM-CP^-met^ to ADPKD kidneys.

To that end, our group reported on the development of peptide amphiphile micelles that were decorated with kidney-targeting peptides including (Lys–Lys–Glu–Glu–Glu)_3_–Lys ((KKEEE)_3_K), which binds megalin, a multiligand receptor highly expressed on renal tubular cells (20–33). Although kidney-targeting peptide amphiphile micelles (KMs) homed to the kidneys in vivo upon intravenous (IV) injection, IV administration is not practical for chronic diseases such as ADPKD that progress over a lifetime (34, 35). To meet the unmet challenges required for inherited and lifelong diseases and improve the bioavailability of drugs for chronic kidney diseases such as ADPKD, herein, we developed a biomaterials-based strategy for oral administration of kidney-targeting nanoparticles and hypothesized that encapsulating KMs loaded with metformin (KM^-met^) into chitosan particles (CP) would enable enhanced nanoparticle bioavailability and drug delivery to the kidneys (Scheme 1) (36, 37). We chose chitosan for developing our oral delivery strategy as chitosan is a biocompatible biomaterial that is naturally derived from crustacean shells and has been reported to improve the drug bioavailability upon oral administration due to its high stability in acidic conditions, like that of the stomach, and its mucoadhesive properties that enable high absorption in the intestines (38–40).

To test our hypothesis, we first incorporated metformin into KMs (KM^-met^) and tested the therapeutic effects of KM^-met^ in vitro. Then, intravital imaging was used to evaluate the mechanism of KM access to the kidneys, and pilot studies in a porcine model were used to study the kidney-targeting ability of KM across animal species. Next, we incorporated KM^-met^ into CP and studied the ability of KM-CP^-met^ to cross an in vitro intestinal model and induce a therapeutic response on renal cells. Finally, we evaluated the pharmacokinetic properties of KM-CP^-met^ in vivo and the therapeutic outcomes in a slow and progressive murine model of ADPKD that mimics the chronic nature of human disease. Overall, we present for the first time an oral delivery nanomedicine strategy designed for PKD and provide evidence for its practical implementation in chronic kidney diseases.

## Results

### Synthesis, characterization, and in vitro therapeutic effects of KM^-met^

**Fig. 1.**
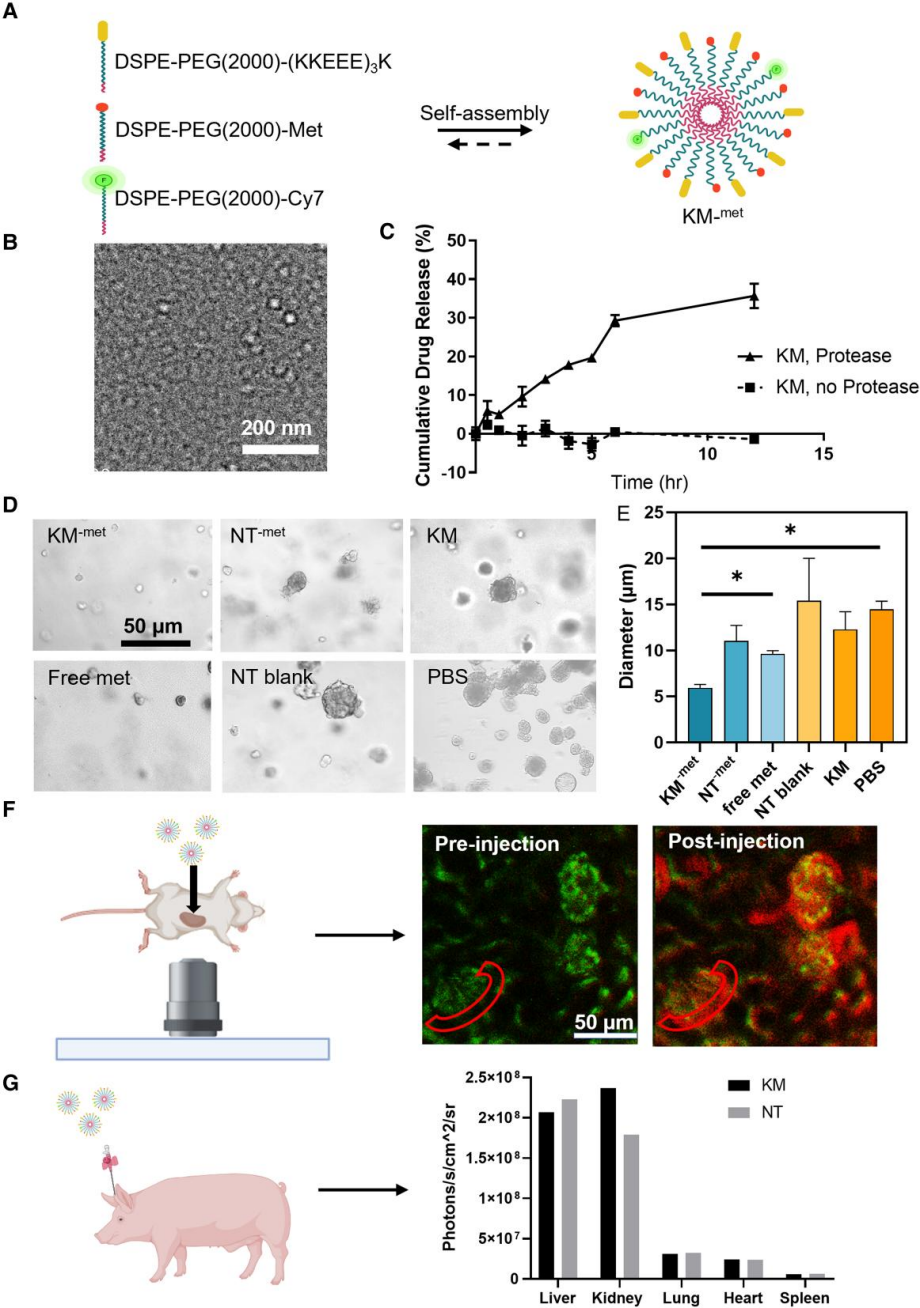
Characterization and in vitro therapeutic effects of KM^-met^. A) Schematic of KM^-met^ self-assembly. B) TEM confirms spherical morphology and the size of KM^-met^ to be approximately 15 nm. C) Steady drug release of metformin from KM over time via protease cleavage. D and E) Brightfield images and quantification of cystogenesis using *Pkd1* null cells treated with KM^-met^, free metformin, NT^-met^, NT blank, KM, or PBS (*P ≤ 0.05, *N* ≥ 4). F) Intravital images of kidney glomeruli after KM micelles were injected into the carotid artery of C57BL/6 mice shows KMs pass the GFB and enter the Bowman's space. G) Pilot study quantifying biodistribution of KM micelle and NT micelle injected into a porcine model, confirming the kidney-targeting ability of KMs across animal species (*N* = 1).

To first construct kidney-targeting micelles (KMs), the kidney-targeting peptide (KKEEE)_3_K, metformin, and the fluorophore Cy7 was conjugated to DSPE-PEG(2000), purified via high performance liquid chromatography (HPLC) and MALDI-TOF/TOF (Fig. S1), and self-assembled under aqueous conditions (Fig. 1A). To characterize the size and shape of KM^-met^, we imaged KM^-met^ by TEM and found the particles were monodispersed and of spherical morphology (Fig. 1B). Next, to verify that free metformin can be released from micelles intracellularly, a commercially available cocktail of proteases derived from *Streptomyces griseus* were incubated with KM^-met^ (41). After 12 h of incubation, a steady release of metformin from KMs was found (35.7 ± 3.2%; release rate of 3 %/h), while no appreciable drug release was found in the absence of proteases (Fig. 1C), confirming that the peptide bond between DSPE-PEG(2000) and metformin was cleaved by proteases as expected (42).

The in vitro therapeutic effects of KM^-met^ were assessed *via* a 3D cyst model (Fig. 1D). Specifically, *Pkd1* null cells generated from the *Pkd1flox/-:TSLargeT* mouse model of ADPKD were cultured in Matrigel and treated with KM^-met^, NT^-met^, or free metformin at a drug concentration of 300 µM and the effects on cyst growth were measured on day 8. Empty nontargeting (NT) micelles, KM, and PBS without metformin also served as additional controls. Quantification of the cyst images showed that the KM^-met^-treated group had significantly smaller cyst diameter (5.9 ± 0.9 µm) when compared to the PBS-treated group (9.6 ± 0.5 µm, *P* ≤ 0.05), which confirmed the therapeutic response of KM^-met^ in vitro (Fig. 1E). In addition, as found in Fig. S2, KM^-met^ showed the highest phosphorylated AMPK to total AMPK ratio among all the metformin-containing groups: 1.1 ± 0.2 for KM^-met^, 0.7 ± 0.01 for NT^-met^, and 0.6 ± 0.04 for free metformin (*P* ≤ 0.05), which confirmed that kidney-targeting micelles improved in vitro delivery of metformin and activation of AMPK (Fig. S2). To validate the megalin-targeting ability of KM, both KM and NT micelles were incubated with *Pkd1* null cells and KM micelles was found to have significantly higher colocalization (0.12 ± 0.02) with the megalin channel as compared to the NT micelles (0.07 ± 0.03, *P* ≤ 0.05, Fig. S3).

Towards in vivo application, intravital imaging was performed to study the mechanism of KM entry and access to the kidneys in real-time by evaluating glomerular filtration and nephron uptake. Under anesthesia, mice were injected with KMs into the canulated carotid artery while placed under an inverted microscope with exposed kidneys. Figure 1F and Video S1 shows KMs enter the glomerulus, pass the glomerular filtration barrier (GFB), and access the Bowman's space (red region) and tubules upon administration. This is in contrast to free amphiphilic monomers (Cy7-DSPE-PEG2000-(KKEEE)_3_K) that showed minimal uptake into the nephron. KM micelles showed significantly higher fluorescence in the Bowman's space as compared to monomers (5.65 × 10^5^ ± 4.8 × 10^4^ a.u. vs. 1.6 × 10^5^ ± 2.4 × 10^4^ a.u. *P* ≤ 0.0001, Figs. 1F and S3, Videos S1 and S2), suggesting the nanoparticle structure is critical for kidney targeting. Additionally, to further probe the kidney-targeting ability beyond mice, we conducted a pilot study and administrated KMs into a porcine model. Upon ex vivo imaging and analysis at 24-h postinjection, KMs were found to have significantly higher kidney/liver ratio (1.14) as compared to NT micelles (0.81) (Figs. 1G and S4), consistent with earlier studies in mice (22). Compared to other nanoparticles that have been proposed for kidney-targeting (Table 1), KMs demonstrate unprecedented kidney-targeting vs. liver uptake, validating the feasibility of enriching drugs to the kidneys using nanoparticle carriers, which has been difficult and limited to date. Additionally, porcine organs including the kidney, liver, heart, lung, and spleen were stained for H&E to evaluate tissue morphology (Fig. S5). As shown in Fig. S5, there was no observable tissue damage within these organs as compared to PBS-treated pigs, confirming the biocompatibility of KMs across animal species.

**Table 1.** Kidney/liver accumulation ratio of various nanoparticles proposed for kidney drug delivery shows KMs have superior targeting.

Kidney/Liver ratio	Nanoparticle	Reference
1.25	KM^a^ in mice	(22)
1.14	KM^a^ in pig	—
0.85	Albumin-based NP	(43)
0.47	Iron oxide NP	(44)
0.29	Quantum dots	(45)
0.05	Gold NP	(46)
0.2	Gold-PEG NP	(47)

^a^Active kidney targeting via (KKEEE)_3_K peptide.

### Loading of KMs within CP

**Fig. 2.**
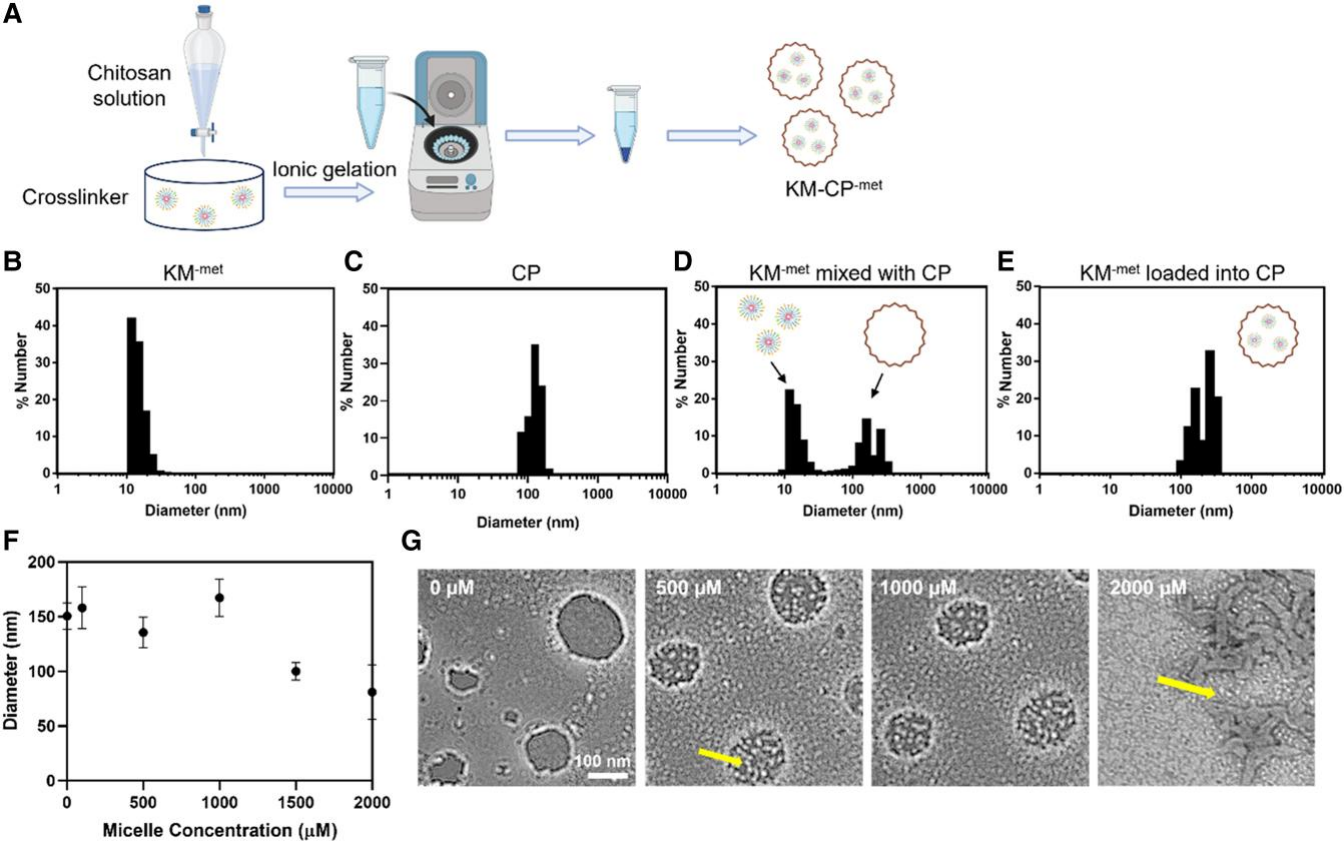
KM loading into CP. A) Schematic of KM^-met^ loading into CP via ionic gelation. Representative DLS measurements of B) KM^-met^, C) CP, D) KM^-met^ mixed with CP, and E) KM_-_^met^ loaded into CP. Micelle peaks corresponding to ∼14 nm were present (D) in the mixed condition but were not seen (E) in the loaded condition. F) DLS measurements of CP loaded with various initial starting concentrations of KM^-met^. G) TEM images of CP synthesized with varying concentrations of micelles (arrows).

Towards application in the context of chronic kidney diseases, we loaded KM^-met^ into CP in order to enhance KM bioavailability and delivery to the kidneys upon oral delivery. We first mixed KM-^met^ into the anionic poly-L-glutamic acid cross-linker solution. The chitosan solution was then added to the cross-linker/micelle solution to form KM-CP^-met^ through ionic gelation (Fig. 2A) (48–50). CPs loaded with KM-^met^ were found to be ∼160 nm in diameter using dynamic light scattering (DLS). As found in Fig. 2B and C, KMs on their own are 13.9 ± 1.8 nm in diameter and CPs are 155.9 ± 14.8 nm as measured by DLS. When micelles were mixed with CPs separately, two distinct size distributions (15.0 ± 2.1 nm and 148.2 ± 17.9 nm) can be observed, which correspond to the expected micelle size of ∼14 nm and the CPs of ∼160 nm (Fig. 2D). However, when micelles are loaded into CPs, the micelle only peak of ∼14 nm is no longer observed, confirming that KMs are successfully loaded into CPs (Fig. 2E).

Next, we probed the maximum micelle loading that would result in stable CP. As found in Fig. 2F, micelle concentrations added to the cross-linker solution of up to 1000 µM had similar sizes to that of unloaded CP (∼160 nm). Beyond 1,000 µM micelle concentration, the diameter of the CP decreases while polydispersity increases, showing a loss of stability. As also confirmed by TEM in Fig. 2G, individual micelles are found encased within CP up to 1,000 µM KM^-met^. However, at 2,000 µM KM^-met^, CP are no longer present and instead, a disordered structure with the majority of micelles existing separately from the bulk chitosan material is observed (arrows, Fig. 2F). Thus, we concluded that a maximum of 1,000 µM micelles can be loaded into CP.

### KM^-met^ release from CP and KM-CP^-met^ therapeutic effects in intestinal and in vitro kidney models

**Fig. 3.**
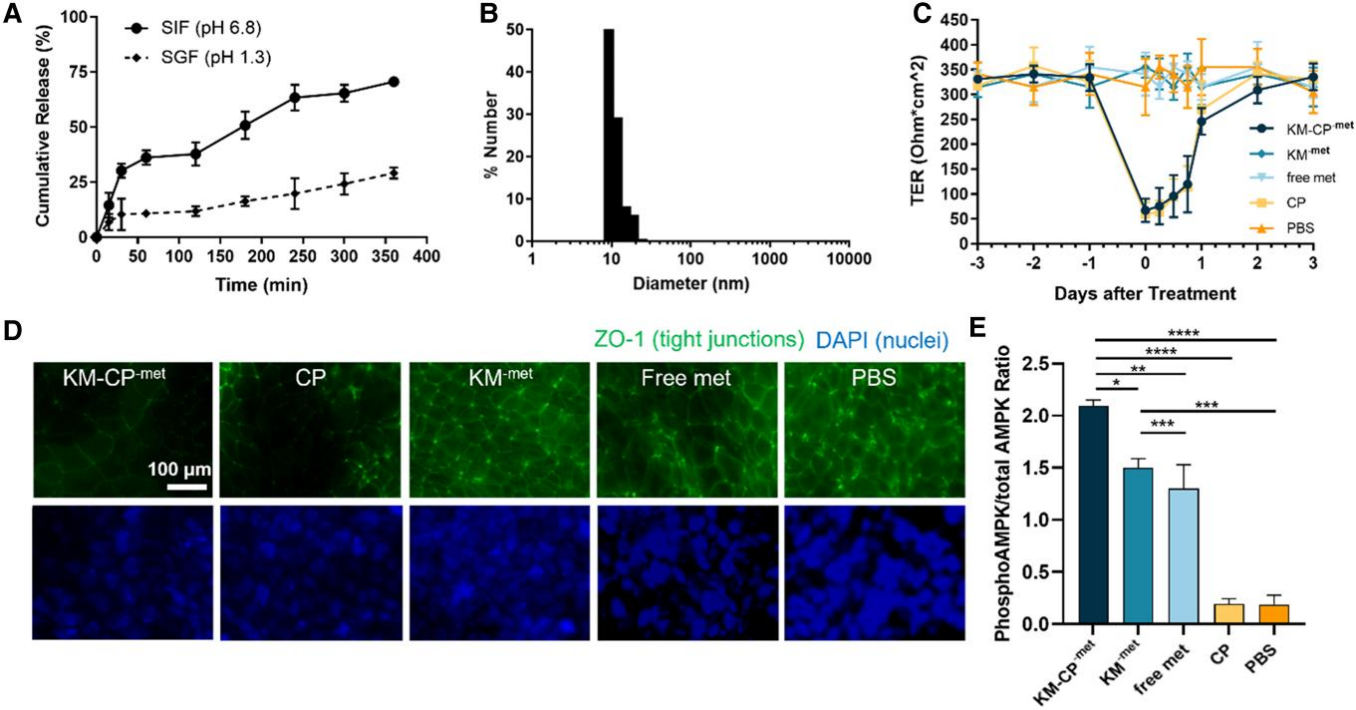
In vitro therapeutic efficacy of KM-CP^-met^. A) In vitro release of KM from KM-CP under pH conditions found in the GI tract (pH = 1.3, SGF; 6.8 SIF, *N* ≥ 4). B) DLS size measurements of micelles released from SIF at 3 h. C) TER measurements of Caco-2 monolayers incubated with KM-CP^-met^, KM^-met^, free metformin, CP, or PBS for up to 6 days. D) Caco-2 monolayers stained with ZO-1 and DAPI at 6 h showed a decrease in ZO-1 signal in treatment groups that contained chitosan. E) Phosphorylated AMPK to total AMPK obtained via ELISA on mpkCCD_c14_ cells to test the therapeutic efficacy of KM-CP^-met^ (*P ≤ 0.05, **P ≤ 0.01, ***P ≤ 0.001, ****P ≤ 0.0001, *N* ≥ 3).

As previously reported, chitosan is stable in low pH but can degrade under neutral pH similar to the intestinal environment, which is favorable for drug absorption into the bloodstream upon oral delivery (36). To test the stability of KM-CP^-met^ under a low pH environment as in the stomach, we incubated KM-CP^-met^ in simulated gastric fluid (SGF, pH = 1.3). After 6 h, 29.1 ± 2.5% of total KM-^met^ was found to be released. This is in contrast to KM-CP^-met^ incubation in simulated intestinal fluid (SIF, pH = 6.8), which released 70.6 ± 1.5% of KM^-met^ (*P* ≤ 0.01, Fig. 3A). To validate whether the released KM^-met^ from CP under neutral pH were intact micelles or disassembled amphiphiles, we conducted DLS measurements at the final timepoint and found intact micelles of ∼14 nm size were released into the supernatant (Fig. 3B). In addition, KM^-met^ released from CP after 12 h were separated via centrifuge and further characterized via DLS along with KM-CP^-met^ to confirm the successful formation of the micelle-in-chitosan structure (Fig. S7).

In addition to protection against an acidic environment mimicking the stomach, chitosan has been reported to open tight junctions within the intestinal epithelium and increase bioavailability in vivo (51). To verify this effect, transepithelial resistance (TER) was measured on human colorectal Caco-2 cells cultured on Transwell permeable supports following treatment with KM-CP^-met^ (52, 53). TER was measured three days before treatment and again after administration of nanoparticles or free drug for up to 3 days. Within 6 h postadministration of KM-CP^-met^, a 79.9% reduction in resistance was observed (from 334.0 ± 26.8 Ohm*cm^2^ to 67.1 ± 23.3 Ohm*cm^2^). On the other hand, no changes in TER were observed for samples treated with KM-^met^, free metformin, or PBS (Fig. 3C) (36). Notably, a recovery to pretreated baseline resistance levels (335 Ohm*cm^2^) was observed 3 days after KM-CP^-met^ treatment, demonstrating that these effects on tight junctions are transient (54). Additionally, we observed a decrease in ZO-1 signal in treatment groups that contained chitosan at 6 hours after treatment (Figure 3D), suggesting that KM-CP^-met^ can enhance penetration through the intestinal epithelium consistent with previous reports (36). Overall, these results using in vitro models show evidence that KM-CP^-met^ has the potential to protect cargo under acidic conditions similar to the environment of the stomach, enhance paracellular transport across the intestinal epithelium, and enable micelle-drug release, thereby promoting higher absorption and bioavailability in circulation (55).

To test the delivery and therapeutic efficacy of KM-CP^-met^ through oral administration, we first utilized a simple, intestine-kidney in vitro model that seeded Caco-2 intestinal cells on the apical side of the Transwell membrane and mpkCCD_c14_ cells grown on the bottom of the plate. After 12 hours, we found that mpkCCD_c14_ cells in the KM-CP^-met^ treatment group showed the highest phosphorylated AMPK to total AMPK via enzyme-linked immunosorbent assays (ELISA), among all met-containing groups: 1.3 ± 0.4 for free metformin, 1.5 ± 0.2 for KM^-met^, and 2.10 ± 0.1 for KM-CP^-met^ (*P* ≤ 0.005), while no change was found for CP and PBS treatment (Fig. 3E). This confirms that the in vitro delivery of metformin and activation of AMPK can be improved through the combined chitosan and kidney-targeting platforms and is the greatest in KM-CP^-met^ group as compared to all other groups (Figure 3E).

### In vivo biodistribution of KM-CP^-met^

**Fig. 4.**
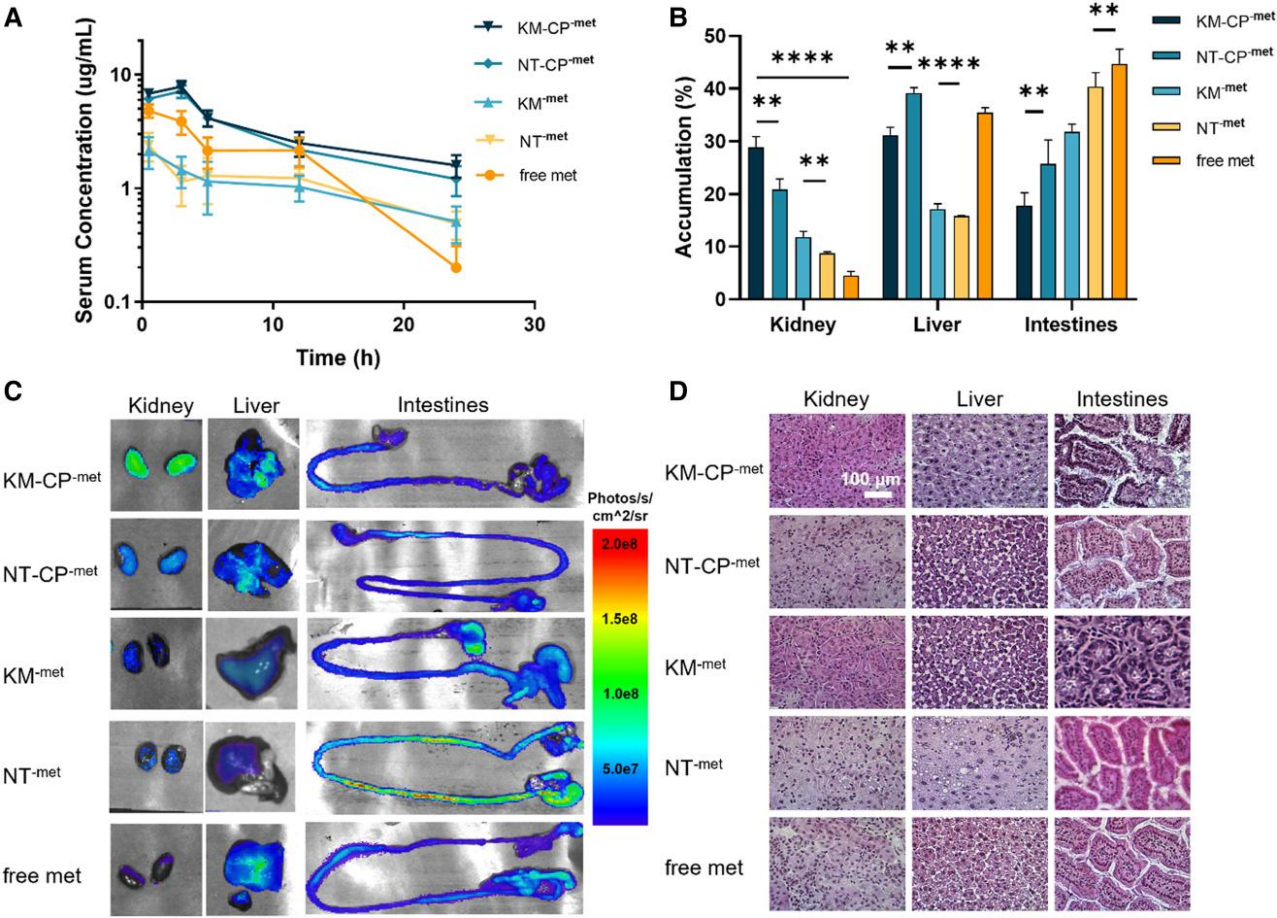
Bioavailability, biodistribution, and biocompatibility of Cy7-labeled KM-CP^-met^ 24 h after oral gavage. A) Serum level of Cy7-labeled micelles in KM-CP^-met^ is the highest after oral delivery. B and C) Quantification of ex vivo organ Cy7 fluorescence levels show highest accumulation in the kidneys for KM-CP^-met^ and NT-CP^-met^. D) H&E staining of organs shows no significant tissue damage or changes in morphology after all treatments (**P ≤ 0.01, ****P ≤ 0.0001, *N* = 4).

To evaluate the ability of CP to enhance micelle bioavailability *via* oral delivery in vivo, wildtype C57BL/6J mice were orally administered Cy7-labeled KM-CP^-met^. Over 24 h, KM-CP-^met^ treated mice consistently showed higher serum fluorescence as compared to NT-CP^-met^, KM^-met^, NT^-met^ and free metformin, demonstrating enhanced bioavailability and depot into systemic circulation (Fig. 4A). Upon ex vivo imaging and analysis, Cy7-labeled KM-CP^-met^ also showed higher kidney accumulation (28.9 ± 4.1%) when compared to NT-CP^-met^ (20.9 ± 4.1%, *P* ≤ 0.01, Figs. 4B and C, S8). Similarly, Cy7-labeled KM^-met^ demonstrated higher renal accumulation (15.9 ± 2.1%) when compared to NT^-met^ (8.7 ± 0.6%, *P* ≤ 0.01), confirming the benefits of CP in enhancing bioavailability through oral administration and the renal targeting ability through the (KKEEE)_3_K peptide in vivo. Immunohistochemistry evaluating the colocalization of nanoparticles and megalin showed 3.5-fold (*P* ≤ 0.05) higher colocalization in mice treated with KM-CP^-met^ as compared to KM^-met^, which confirmed CP improved the bioavailability of KM^-met^ upon oral administration, thus leading to higher KM^-met^ targeting and delivery to the kidneys (Fig. S7).

Additionally, mice organs including the brain, lung, heart, liver, spleen, intestine, kidneys, and bladder were stained for H&E in order to evaluate tissue morphology post-treatment (Figs. 4D and S8). As shown in Fig. 4D, there was no observable tissue damage in any of the organs, including the kidneys, liver, and intestines where nanoparticles showed higher accumulation confirming biocompatibility of all groups.

### Therapeutic efficacy of KM-CP^-met^ in ADPKD mice

**Fig. 5.**
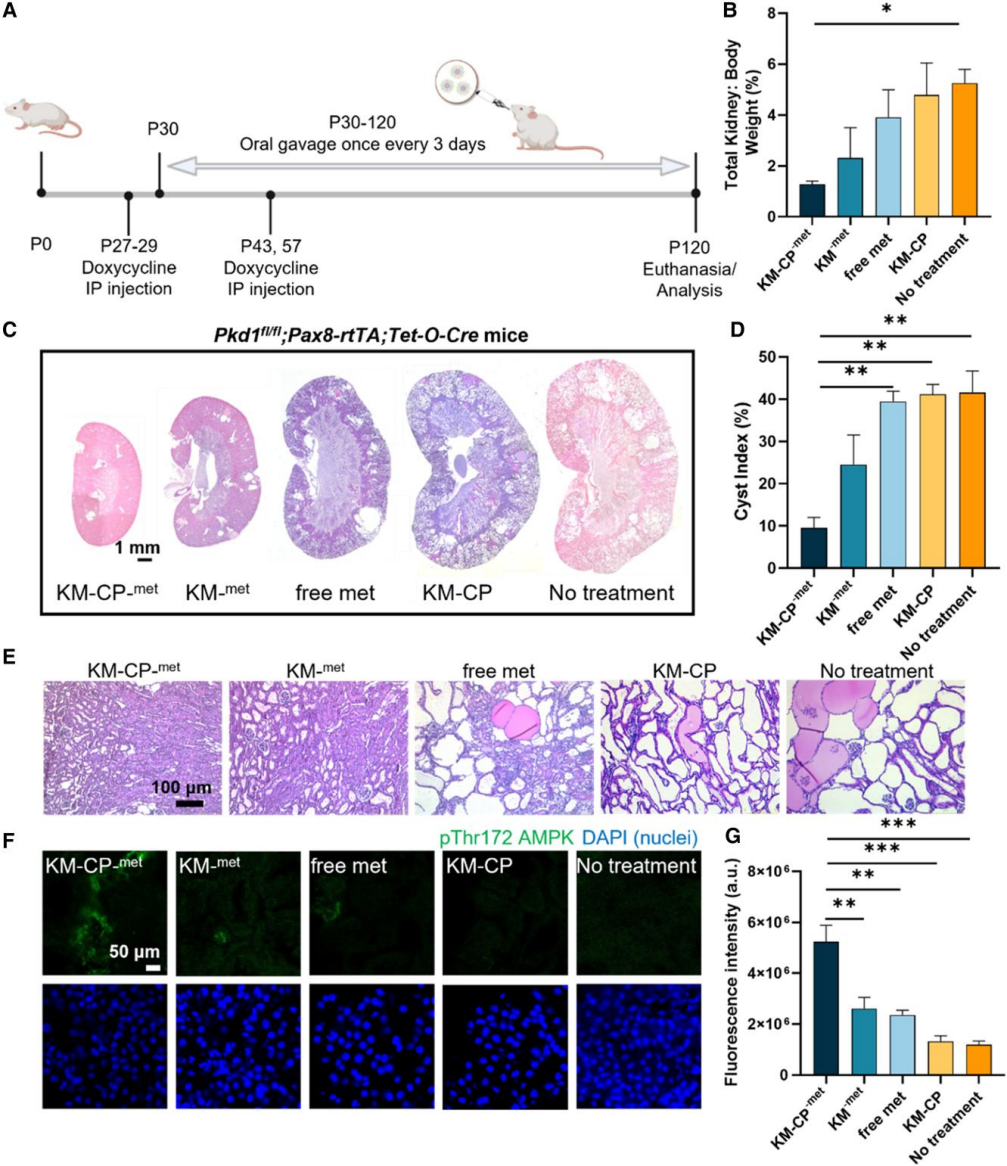
Therapeutic efficacy of orally administered KM-CP^-met^ in ADPKD mice. A) Timeline of *Pkd1^fl/fl^*;*Pax8-rtTA*;*Tet-O-Cre* mice treatment. B) The lowest KW/BW ratio was also found in the KM-CP^-met^ treated group. C) H&E staining of whole kidneys shows smallest kidneys with fewer cysts in the KM-CP^-met^ treatment group. D) Cystic index of the KM-CP^-met^ treated group is significantly decreased as compared to KM^-met^ and no treatment groups. E) H&E staining of kidney tissues shows smaller cyst size and fewer cysts in the KM-CP^-met^ treatment group. F) Immunohistochemistry and G) quantitative analysis of pThr172 AMPK in kidney sections and quantitative analysis shows significantly higher pThr172 AMPK fluorescence intensity of mice treated with KM-CP^-met^ as compared to other treatment groups (*P ≤ 0.05, **P ≤ 0.01, ***P ≤ 0.001, *N* ≥ 3).

The therapeutic effects of KM-CP^-met^ upon oral administration was tested in a slowly progressing ADPKD murine model using *Pkd1^fl/fl^*;*Pax8-rtTA*;*Tet-O-Cre* mice, which mimics the chronic nature of the human disease (56). In this model, PKD is developed by conditional knockout of the *Pkd1* gene and induced by doxycycline injection starting on postnatal day 27 (P27). After *Pkd1* knockout, mice were orally administered KM-CP^-met^, KM^-met^, free metformin, or KM-CP every three days at a metformin concentration of 300 mg/kg/day (Fig. 5A), as reported previously in ADPKD preclinical studies (15, 17, 36), and euthanized on P120 when severe disease is expected (57). As found in Fig. 5B, KM-CP^-met^-treated mice showed a lower kidney weight/body weight (KW/BW) ratio (1.3 ± 0.3%) as compared to other treatment groups and the no treatment control (5.4 ± 0.6%, *P* ≤ 0.05, Fig. 5B). The KW/BW level of healthy mice is around 1% (58), which confirmed that *Pkd1*-knockout mice under KM-CP^-met^ showed healthy kidney weight. In addition, mice treated with KM-CP^-met^ showed a smaller kidney size (Fig. 5C) and a decreased cystic index (9.6 ± 4.2%, i.e. the percentage of cystic area divided by total kidney area) as compared all other groups (Fig. 5D and E). In addition, KM-CP^-met^-treated mice were found to have significantly higher pThr172 AMPK levels as compared to all other control groups, confirming the activation of pThr172 AMPK by KM-CP^-met^ (Fig. 5F and G).

Importantly, the long-term, repeated treatment, as often required in chronic diseases, of KM-CP^-met^ did not alter serum electrolyte concentrations, which were found to remain similar for all groups. In addition, blood urea nitrogen (BUN) levels of KM-CP^-met^ treated mice were within the healthy mice BUN range (∼40 mg/dL) (59). Thus, these results indicate that KM-CP^-met^ has high biocompatibility and is safe despite continued exposure over four months (Table 2). In summary, KM-CP^-met^ is a viable nanomedicine strategy for ADPKD and is the first development of an oral delivery strategy of kidney-targeting nanoparticles for chronic kidney diseases.

**Table 2.** Serum components, electrolytes, and kidney health markers of ADPKD mice upon treatment.

	Na (mmol/L)	K (mmol/L)	Cl (mmol/L)	iCa (mmol/L)	tCO2 (mmol/L)	Glu (mmol/L)	BUN/Urea (md/dL)	Crea (md/dL)	Hct (PCV)	Hb (g/dL)
KM-CP^-met^	132.3 ± 7.5	7.5 ± 2.2	120.0 ± 7.1	1.2 ± 0.1	16.7 ± 4.5	344.0 ± 24.0	34.0 ± 10.8	<0.2	41.7 ± 0.5	14.2 ± 0.2
KM^-met^	146.7 ± 1.9	4.9 ± 0.2	114.0 ± 2.8	1.1 ± 0.1	25.0 ± 2.2	229.7 ± 32.3	38.3 ± 19.7	<0.2	36.3 ± 4.9	12.4 ± 1.7
Free met	144.6 ± 1.6	5.9 ± 1.4	113.0 ± 3.2	1.1 ± 0.2	23.1 ± 0.8	219.6 ± 24.4	22.8 ± 7.8	<0.2	37.4 ± 3.0	12.8 ± 0.9
KM-CP	143.5 ± 5.5	5.8 ± 1.7	114.4 ± 4.0	1.2 ± 0.1	22.4 ± 3.9	188.9 ± 22.5	34.3 ± 19.1	<0.2	35.9 ± 2.8	12.2 ± 1.0
No treatment	145.0 ± 1.6	5.3 ± 0.2	113.3 ± 3.3	1.2 ± 0.1	21.8 ± 1.8	239.3 ± 31.1	24.3 ± 1.8	<0.2	36.3 ± 3.9	12.3 ± 1.3

## Discussion

Over the past few decades, the development of nanomedicine has emerged as a promising approach for the treatment of various diseases. Although more than 50 pharmaceutical nanoformulations have been approved by the FDA for clinical use (60), the development of nanomedicine for the treatment of chronic kidney diseases like ADPKD is limited. To our knowledge, KMs loaded into CP is the first development of an oral delivery nanoformulation for kidney disease. Specifically, we incorporated metformin into KMs and showed that KM^-met^ enhanced renal cell targeting and therapeutic efficacy by reducing cyst growth (Figure 1). We also evaluated the mechanism of KM access into the kidneys via intravital imaging and found KMs significantly enter the Bowman's space, while free amphiphilic monomers do not reach the kidneys. This may be due to monomer binding to serum components such as albumin, which do not pass the GFB (28, 61). Overall, these results show evidence that after depot into the bloodstream, the nanoparticle structure is critical for transport into the kidneys and that KMs passage through the GFB and access the renal tubular system, which enable drug delivery in kidney diseases such as ADPKD.

Notably, nanoparticle design for kidney drug delivery has been a long-standing challenge in the field due to the lack of nanoparticle kidney retention via clearance into urine (e.g. 10 nm iron oxide nanoparticles (44) and 6 nm gold nanoparticles (46)) or due to nonspecific protein absorption which can lead to protein corona formation and an growth in nanoparticle size that leads to liver uptake, In contrast, KMs demonstrate remarkable kidney-targeting performance in both mice and porcine models (Fig. 1H). This may be due to the zwitterionic properties of the megalin-binding peptide, (KKEEE)_3_K, in addition to PEGylation, which hinder serum protein adsorption of nanoparticles and allows KMs to maintain their small size of <12–15 nm for GFB penetration (22). This exceptional performance positions KM as an ideal candidate for delivering small molecular drugs directly to the kidneys.

As many kidney diseases are chronic kidney diseases that last a lifetime, oral delivery is the most practical and patient-friendly route of administration. KM^-met^ loading into CP (Fig. 2) enabled protection of the cargo under gastric pH while also opening tight junctions to enhance paracellular transport and bioavailability of KM^-met^ upon its release (Fig. 3), consistent with earlier results (36). Of note, the bioavailability and biodistribution data shown in Fig. 4 indicate that KM-drug loading into chitosan may afford less frequent and/or lower drug dosing to achieve the same efficacy. Then, upon access to circulation, the selectively enhanced kidney accumulation observed with our kidney-targeting nanoparticles should also result in fewer off-target side effects and enhanced tolerability of metformin or other drugs used to treat ADPKD and other kidney diseases. In addition, tissue morphology of mice organs further confirmed the high biocompatibility of KM-CP^-met^ (Fig. 4). It is noteworthy that NT-CP^-met^ exhibited the second highest renal accumulation among all groups, reaffirming the superior function of CP in enhancing the bioavailability of micelles. Our previous study demonstrated that NT micelles accumulated in the kidneys following IV injection (25), and this aligns with the high renal accumulation observed for NT-CP^-met^ upon oral administration in the current study. Ultimately, we demonstrate that both the chitosan and targeting micelles were critical for oral administration and kidney accumulation, which conferred greater therapeutic benefits in ADPKD mice (Fig. 5). In our previous study by Wang et al., metformin-loaded chitosan nanoparticle system demonstrated a 23% decrease in KW/BW and a 13% decrease in cystic index as compared to free met-treated group. However, KM-CP^-met^ exhibited a remarkable 69% decrease in KW/BW and a substantial 75% decrease in cystic index as compared to free met-treated group. Despite using different PKD animal model, the data consistently indicates that KM-CP^-met^ exhibits a superior therapeutic effect as compared to metformin-loaded chitosan nanoparticle.

The primary obstacle in the current management of kidney disease lies in the inadequate drug concentration within the kidney. This limitation is particularly evident in the case of ADPKD, where the only FDA-approved treatment, tolvaptan, achieves ∼5.7% accumulation in the kidney after oral administration (62). Similarly, in our study, ∼4.5% of free metformin accumulated in the kidney. Given the pronounced deficiency in kidney bioavailability observed with free drugs, our innovative oral kidney-targeting nanoformulation emerges as a promising solution to the current clinical challenges by providing a precise, efficient, and patient-friendly treatment that enables direct drug delivery to the kidneys and minimizes accumulation in nontarget organs. Although our current study incorporated one ADPKD drug candidate, our biomaterials and nanoparticle-based platform can benefit other candidate ADPKD drugs that also have low oral bioavailability, as well as gene therapy, thereby expanding treatment options for ADPKD and other kidney diseases. Additionally, future studies will concentrate on investigating the pharmacokinetic properties and biodistribution of KM-CP-met utilizing the slow PKD mice model.

In summary, our studies show the therapeutic potential and safety of KM-drugs loaded into CP as a strategy for combined kidney-targeting and oral drug delivery in ADPKD, and this transformative work extends far beyond ADPKD, offering invaluable insights applicable to a broad range of chronic and genetic kidney diseases which can greatly benefit from targeted drug delivery strategies but currently have no nanomedicine options in the clinic.

## Materials and methods

### Synthesis of therapeutic KMs

The (KKEEE)_3_K targeting peptide was synthesized using standard Fmoc-mediated solid phase peptide synthesis methods on rink Amide resin (Anaspec, Fremont, CA, USA) using an automated benchtop peptide synthesizer as previously described (PS3, Protein Technologies, Tucson, AZ, USA) (25). A cysteine was added to the peptide sequence at the N-terminus to allow for a thioester linkage. Peptides were cleaved from the resin and deprotected with 94:2.5:2.5:1 by volume trifluoroacetic acid:1,2-ethanedithiol:H_2_O:triisopropylsilane and were precipitated and washed several times with cold diethyl ether, dissolved in water, lyophilized, and stored as powders at −20°C. Crude, peptide mixtures were purified by reverse-phase HPLC (HPLC, Shimadzu, Kyoto, Japan) on a C8 column (Phenomenex, Torrance, CA, USA) at 50°C using 0.1% formic acid in acetonitrile/water mixtures and characterized by matrix-assisted laser desorption ionization time-of-flight mass spectral analysis (MALDI-TOF/TOF, Autoflex Speed, Bruker, Billerica, MA, USA, Figure S1). Cysteine-containing peptides were conjugated to 1,2-distearoyl-sn-glycero-3-phosphoethanolamine-*N*-methoxy-poly(ethylene glycol 2000) (DSPE-PEG(2000)-maleimide, Avanti Polar Lipids, Alabaster, AL, USA) by adding an equimolar amount of the lipid to peptide in MilliQ water. After gentle mixing for 1 week, the resulting DSPE-PEG(2000)-(KKEEE)_3_K was purified by HPLC on a C4 column as described above.

Fluorophore-conjugated amphiphiles were synthesized by conjugating Cy7 via a peptide bond to DSPE-PEG(2000)-amine (Avanti Polar Lipids, Alabaster, AL, USA) by adding an equimolar amount of Cyanine7 NHS ester (Lumiprobe, Hunt Valley, MD, USA) to the lipid dissolved in 0.1 M aqueous sodium bicarbonate buffer (pH 8.5). After reaction at room temperature and protected from ambient light for 24 hours, the mixture was purified on a C4 column and characterized as described above. Cy7-met was also synthesized via conjugating the ester group of Cy7 to the amine group of metformin hydrochloride as described above.

DSPE-PEG(2000)-met was synthesized by conjugating metformin hydrochloride via a peptide bond to DSPE-PEG(2000)-NHS (Avanti Polar Lipids, Alabaster, AL, USA) by adding a 5× molar excess of metformin to the lipid dissolved in 10 mM aqueous sodium carbonate buffer (pH 8.5). After reaction at room temperature and protected from ambient light for 24 h, the mixture was purified on a C4 column and characterized as described above (Fig. S1).

To construct micelles, the appropriate DSPE-PEG(2000) amphiphiles were dissolved in methanol or chloroform and evaporated under a steady stream of nitrogen. The resulting film was dried under vacuum overnight and hydrated at 80°C with either MilliQ water or PBS, vortexed and sonicated, and allowed to cool to room temperature. Therapeutic KM (KM^-met^ Cy7) were composed of an amphiphile molar ratio of 10:45:45 consisting of DSPE-PEG(2000)-Cy7:DSPE-PEG(2000)-(KKEEE)_3_K:DSPE-PEG(2000)-met. NT-^met^ Cy7 were composed of an amphiphile molar ratio of 10:45:45 consisting of DSPE-PEG(2000)-Cy7:DSPE-PEG(2000)-methoxy:DSPE-PEG(2000)-met.

### Cell culture

Mouse kidney cortical collecting duct (mpkCCD_c14_) cells were expanded in culture media comprised of Dulbecco's Modified Eagle Medium (DMEM)/F12 supplemented with insulin, dexamethasone, selenium, transferrin, triiodothyronine, glutamine, d-glucose, epidermal growth factor, HEPES, sodium pyruvate as described (63). Media was changed every two days and subcultures were passaged every 7–8 days. Human colon epithelial cells (Caco-2, ATCC HTB-37, ATCC, Manassas, VA, USA) were cultured following the manufacturer's recommendations. Cells were expanded in DMEM supplemented with 10% fetal bovine serum (FBS) and 1% penicillin–streptomycin. *Pkd1* null proximal renal tubule cells (*Pkd1* null cells) isolated from *Pkd1flox/-:TSLargeT* mice were cultured in DMEM/F12 media, 2% FBS, 1× Insulin-Transferrin-Selenium, and ∼2 nM of 3,3′,5-Triiodo-L-thyronine sodium salt. Cells were expanded at 37°C in a humidified incubator under 5% CO_2_. Cells at passage 3 were used for studies, and the media were changed every 2–3 days.

### Drug release of metformin from KM^-met^

To assess the rate of metformin release from KM^-met^, 1000 µM of KM^-met^ in PBS was treated with proteases (3.3 U/mL from *S. griseus*, Sigma Aldrich, St. Louis, MO, USA) and placed in Slide-A-Lyzer dialysis cassettes (Thermo Fisher Scientific, Waltham, MA, USA) with a molecular weight cutoff of 2,000 Daltons in 10 mM sodium acetate buffer at pH 7.45. Metformin has a molecular weight of 129.16 g/mol, while DSPE-PEG(2000) and DSPE-PEG(2000)-met has a molecular weight of 2,805 g/mol and 2,895 g/mol, respectively, and are unable to escape the dialysis compartment. Measurements of free metformin release out of the dialysis chamber were made at select timepoints (30 min, 1, 2, 3, 4, 5, 6, and 12 h) by UV-VIS spectrophotometer at an absorbance of 233 nm (Nanodrop, Thermo Fisher Scientific, Waltham, MA, USA).

### Inhibition of cystogenesis in vitro by KM^-met^

Fifty microliter of Matrigel from BD Biosciences was added to each well in a 96 well plate and solidified at 37°C for 15 min. *Pkd1* null cells were trypsinized and resuspended with 150 μL of 2% Matrigel in assay medium to achieve approximately 3,000 cells/well, and the cells were grown for 1–2 days. Cells were then treated with 300 µM metformin in KM^-met^, NT^-met^, or free metformin formulations, along with NT blank, (KKEEE)_3_K only, or PBS for 8 days. On day 8, cysts were imaged (Leica DMi8, Leica, Wetzlar, Germany), and the size of cysts was determined by ImageJ (NIH).

### Phospho-AMPK measurements

To assess therapeutic efficacy of KM^-met^ and KM-CP^-met^ in vitro, the cellular levels of phospho-AMPK (Kit #7959) and total AMPK (Kit #7961) were measured via ELISA (Cell Signaling Technologies, Danvers, MA, USA) according to the manufacturer's instructions. To collect cysts grown in Matrigel, cysts were kept on ice for 1 h to liquidize the Matrigel and centrifuged at 400*×g* for 5 min to collect the cyst in the pellet. All standards and samples were measured on a Varioskan LUX microplate reader at a wavelength of 450 nm. The phospho-AMPK to total AMPK ratio was normalized to the PBS group and presented in percentage.

### In vitro KM binding to megalin

To assess the specific binding of KM micelles to megalin receptor on pkd1 null cells, KM and NT micelles were incubated with pkd1 null cells growing on a coverslip for 15 min. For staining of megalin, cell slides were fixed with 4% paraformaldehyde for 15 min and blocked with 1% BSA for 1 h at room temperature. Sections were then incubated overnight at 4°C in a humidified chamber with an antibody against megalin (Abcam, Cambridge, UK, 1:100). The next day, the cell slides were incubated for 1 h at room temperature using Goat anti-Rabbit IgG Superclonal Recombinant Secondary Antibody, Alexa Fluor 647 (Thermo Fisher Scientific, Waltham, MA, USA, 1:2,000). Nuclei were then counterstained with DAPI and slides were mounted using VectaMount mounting medium (Vector Laboratories, Burlingame, CA, USA). The slides were imaged via Zeiss 880 confocal microscope, and fluorescence intensity was quantified by ImageJ.

### Intravital imaging to evaluate mechanism of kidney access by KM

To study the passage of KM through the GFB, intravital imaging of KMs was performed. Under continuous anesthesia (isoflurane 1–2% inhalant), mice were placed on the stage of the inverted microscope with the exposed kidney mounted in a coverslip-bottomed chamber bathed in normal saline as previously reported (64, 65). Body temperature was maintained with a homeothermic blanket system (Harvard Apparatus, Holliston, MA, USA). Alexa Fluor 488-conjugated Dextran 500 kDa (Thermo Fisher, Waltham, MA, USA) was administered intravenously by retro-orbital injections to label the circulating plasma (30 µL IV bolus from 10 µg/mL stock solution). 100 µL of 30 µM Cy7-labeled-KMs or 3 µM unassembled amphiphiles ((KKEEE)_3_K-Cy7-DSPE-PEG(2000)) were administered into the canulated carotid artery. Note that the volume of blood in a mouse is ∼1.5–2 mL and thus, the 3 µM concentration of amphiphiles in circulation will be approximately 0.15–0.2 µM, which is below the critical micelle concentration of ∼1 µM (66). The images were acquired using a Leica SP8 DIVE multiphoton confocal fluorescence imaging system with a 40× Leica water-immersion objective (numerical aperture [NA] 1.2) powered by a Chameleon Discovery laser at 960 or 1,100 nm (Coherent, Santa Clara, CA, USA) and a DMI8 inverted microscope's external Leica 4Tune spectral hybrid detectors (emission at 510–530 nm for GFP, at 580–640 nm for AF594, and at 720–850 for Cy7, Leica Microsystems, Heidelberg, Germany). The potential toxicity of laser excitation and fluorescence to the cells was minimized by using a low laser power and high scan speeds to keep total laser exposure as minimal as possible. Fluorescence images were collected in volume and time series (xyt, 526 ms per frame) with the Leica LAS X imaging software and using the same instrument settings (laser power, offset, gain of both detector channels).

### Biodistribution of micelles in Yorkshire pig

To study the biodistribution of micelles in large animals, micelles were sent to Recombinetics (Eagan, MN, USA) and injected into 7-week-old Yorkshire pigs. Briefly, 11 mL of 100 µM Cy7-labeled KM micelle, NT micelle or PBS was injected into pig via an ear vein catheter. Pigs were euthanized 24 h postinjection and organs (e.g. heart, lungs, liver, kidneys, and spleen) were excised, imaged ex vivo on an AMI HTX in vivo imaging system, and the fluorescence signal was quantified via Aura software (Spectral Instruments Imaging, Tucson, AZ, USA).

### Synthesis of CP

The assembled micelles were loaded into CP via ionic gelation (36). Chitosan (Heppe Medical Chitosan GmbH, Halle, Germany) with 200 mPas viscosity and 85% degree of deacetylation was dissolved at a 2 mg/mL concentration in a solution of 0.5% acetic acid in MilliQ water. Then, a solution of 1 mg/mL anionic cross-linker of poly-L-glutamic acid sodium salt (Sigma Aldrich, St. Louis, MO, USA) was added as the solvent to a thin film of KM-^met^, then hydrated to form micelles. The chitosan solution was added dropwise under constant stirring to the cross-linker/micelle solution at a volume ratio of 5:2. An opalescent suspension was formed spontaneously. CP were separated by microcentrifugation at 14,000 RPM at 14°C for 30 min. The pellet was then washed with increasing % of ethanol in water and used immediately for studies, or frozen and lyophilized and stored at 4–8°C.

### Characterization of Micelle loading into CP

DLS: CP synthesized with 0–2,000 µM of KM^-met^ in PBS were filtered through Puradisc 0.2-µm polyvinylidene fluoride membrane filters (GE Healthcare Life Sciences, Pittsburgh, PA, USA). DLS measurements were determined at 163.5° and 532 nm using a Wyatt Technology Möbiuζ system (Santa Barbara, CA, USA, *N* ≥ 3). All measurements were carried out at 25°C in MilliQ water after equilibrating for 5 min.

Transmission electron microscopy (TEM): Negatively stained samples for TEM were prepared by placing CP synthesized with 0–2,000 µM of KM^-met^ in MilliQ water on 400 mesh lacey carbon grids (Ted Pella, Redding, CA, USA) for 5 min. Excess liquid was wicked away with filter paper, and the grid was washed with MilliQ water before placing 2 wt.% uranyl acetate solution for 2 min and then washing with MilliQ water. Dried samples were immediately imaged on a JEOL JEM-2100F TEM (JEOL, Ltd., Tokyo, Japan).

### Micelle release from CP

Micelle release studies from CP was performed in SGF composed of 2.0 g/L sodium chloride and 2.9 g/L HCl (pH 1.3) or simulated intestinal fluid (SIF) composed of 0.62 g/L sodium hydroxide and 6.8 g/L potassium phosphate monobasic (pH 6.8) (67). KM^-met^ released from CP was quantified at 233 nm using a NanoDrop One microvolume UV-Vis spectrophotometer for up to 6 h at room temperature (Thermofisher Scientific, Waltham, MA, USA). At the endpoint, the degraded CP were spun down at 14,000*×g* for 10 min, and the supernatant was measured in DLS to verify the presence of intact micelles.

### TER measurement after CP treatment

To test whether KM-CP^-met^ can open tight junctions within the intestinal epithelium, Caco-2 cells were seeded at an initial density of 3 × 10^5^ cell/cm^2^ onto Transwell inserts (Corning, NY, USA; diameter 6.5 mm, growth area 0.33 cm^2^, pore size 0.4 µm) and maintained for 21 days to form a confluent monolayer. TER measurements were performed using an EVOM2 Epithelial Voltohmmeter (World Precision Instruments, USA). TER was measured 3, 2, and 1 day before treatment to establish baseline measurements, then every 6 h immediately after treatment with KM-CP^-met^, CP, free metformin, KM^-met^, or PBS until 24 h, and then again on 2 and 3 days postadministration.

### Pharmacokinetic properties of orally administered KM-CP^-met^

All animal procedures followed NIH guidelines for the care and use of laboratory animals and were approved by the University of Southern California's Institutional Animal Care and Use Committee. To evaluate the biodistribution of KM-CP^-met^, 6- to 7-week-old male and female C57BL/6J mice (Jackson Laboratories, Bar Harbor, ME, USA) were orally gavaged with 200 μL of 500 µM Cy7-labeled KM-CP^-met^, NT-CP^-met^, KM^-met^, NT^-met^, or free met. Mice were euthanized 24 h postinjection and organs (e.g. brain, heart, lungs, liver, kidneys, spleen, intestines, and bladder) were excised, imaged ex vivo on an AMI HTX in vivo imaging system, and the fluorescence signal was quantified via Aura software (Spectral Instruments Imaging, Tucson, AZ, USA, *n* ≥ 4). The mean radiance (photons/s/cm^2^/sr) for each organ was quantified as a region of interest, and % of total organ fluorescence was obtained by dividing the fluorescence level of each organ by the sum of all the organ regions. Background was subtracted from the PBS-treated group.

To calculate half-life, blood draws were performed either retro-orbitally or via tail vein at 30 min, 3, 6, 12, and 24 h postadministration. Fluorescence was measured in serum and quantified using a Cy7 metformin calibration curve developed in mouse serum.

### Histological analysis

Brain, lung, heart, liver, spleen, intestines, kidneys, and bladder were harvested from the euthanized mice. Immediately after imaging, organs were flash frozen in OCT and sectioned into 10 µm thick slices via a CM3050S cysostat (Leica CM3050S, Leica, Wetzlar Germany). Tissue samples were stained with H&E and imaged (Leica DMi8, Leica, Wetzlar, Germany).

### Immunohistochemistry

For staining of megalin and AMPK, kidney sections were fixed with 4% paraformaldehyde for 15 min, permeabilized with 0.25% Triton X-100 for 10 min and blocked with 5% BSA for 1 h at room temperature. Sections were then incubated overnight at 4°C in a humidified chamber with an antibody against megalin (Abcam, Cambridge, UK, 1:100) or Phospho-AMPK alpha-1,2 (Thr183, Thr172) (Thermo Fisher Scientific, Waltham, MA, USA, 1:100). The next day, kidney sections were incubated for 1 h at room temperature using Goat anti-Rabbit IgG Superclonal Recombinant Secondary Antibody, Alexa Fluor 488 (Thermo Fisher Scientific, Waltham, MA, USA, 1:2,000). Nuclei were then counterstained with DAPI and slides were mounted using VectaMount mounting medium (Vector Laboratories, Burlingame, CA, USA). Fluorescence intensity was quantified by ImageJ.

### Therapeutic efficacy of KM-CP-^met^ in ADPKD mice

To assess the ability of CP to enhance the therapeutic efficacy of orally administered KM-^met^ in ADPKD, KM-CP^-met^, or KM^-met^ (300 mg/kg met) was administered in *Pkd1^fl/fl^*;*Pax8-rtTA*;*Tet-O-Cre* mice, which mimics the slowly progressing ADPKD similar to the human condition. Mice were IP injected with doxycycline (50 mg/kg/day) on postnatal day 27–29 (P27–P29), P43, and P57 before they were orally gavaged every 3 days starting on P30 until P120. On P120, kidneys were excised to assess kidney weight to body weight (KW/BW) ratio and were H&E stained measure cystic index. Cystic index is defined as the percentage of cystic area divided by total kidney area (68) and was measured by ImageJ.

### Kidney health in ADPKD mice

On the day of harvest, 90 µL of blood taken from the submandibular vein was analyzed using Chem-8+ cartridges for the i-Stat Handheld Blood Analyzer (Abbott, Chicago, IL, USA). Kidney health markers including measurements of plasma sodium (Na), potassium (K), chloride (Cl), ionized calcium (iCa), total carbon dioxide (tCO2), glucose (Glu), BUN/urea, creatinine (Crea), hematocrit (Hct), and hemoglobin (Hb) were evaluated.

### Statistical analysis

A Student's *t*-test was used to compare means of pairs using GraphPad Prism 8 (San Diego, CA, USA). ANOVA with a Tukey's test for post hoc analysis was used to determine statistical significance, and *P* ≤ 0.05 was considered to be significant.

## Supplementary Material

pgae187_Supplementary_Data

## Data Availability

All data are included in the manuscript and/or supporting information.
