# Supplementary material for: Oral delivery of nanomedicine for genetic kidney disease
Source: PNAS Nexus. 2024 May 10;3(5):pgae187. doi: 10.1093/pnasnexus/pgae187 (PMC11131023; doi:10.1093/pnasnexus/pgae187)
Supplement: pgae187_Supplementary_Data [file pgae187_supplementary_data.zip › PNASNEXUS-PNASNEXUS-2023-01030-TRR-s03.docx]

Oral Delivery of Nanomedicine for Genetic Kidney Disease

Yi Huang^a,^ ^‡^, Jonathan Wang^a,^ ^‡^, Valeria Mancino^b,c^, Jessica Pham^b,c^, Colette O’Grady^a^, Hui Li^b,c^, Kairui Jiang^a^, Deborah Chin^a^, Christopher Poon^a^, Pei-Yin Ho^b,c^, Georgina Gyarmati^d^, János Peti-Peterdi^d^, Kenneth R. Hallows^b,c^ and Eun Ji Chung^a,b,e,f,g,h,i^.

^a^Department of Biomedical Engineering, University of Southern California, Los Angeles, CA, USA

^b^Department of Medicine, Division of Nephrology and Hypertension, Keck School of Medicine, University of Southern California, Los Angeles, CA, USA

^c^USC/UKRO Kidney Research Center, Keck School of Medicine, University of Southern California, Los Angeles, CA, USA

^d^Department of Physiology and Neuroscience, and Medicine, Zilkha Neurogenetic Institute, University of Southern California, Los Angeles, CA, USA

^e^Department of Chemical Engineering and Materials Science, University of Southern California, Los Angeles, CA, USA

^f^Department of Surgery, Division of Vascular Surgery and Endovascular Therapy, Keck School of Medicine, University of Southern California, Los Angeles, CA, USA

^g^Department of Stem Cell Biology and Regenerative Medicine, University of Southern California, Los Angeles, CA, USA

^h^Norris Comprehensive Cancer Center, University of Southern California, Los Angeles, CA, USA

^i^Bridge Institute, University of Southern California, Los Angeles, CA, USA

*Corresponding author: Eun Ji Chung

University of Southern California

Department of Biomedical Engineering

1002 Childs Way, MCB 357

Los Angeles 90089, CA, USA

Tel: 1 213 740 2925

Email: eunchung@usc.edu

**Supplementary Information**


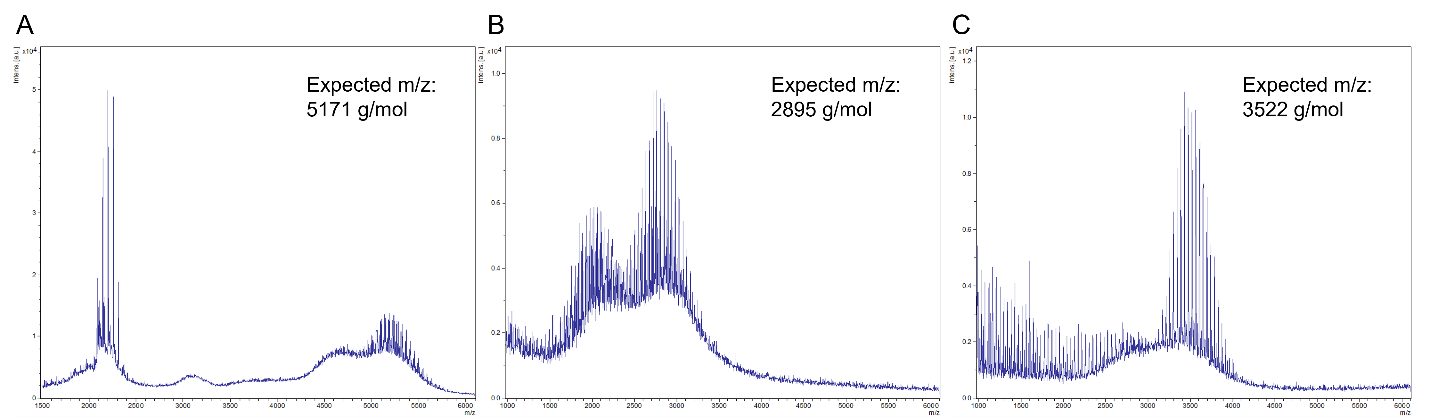


Figure S1. MALDI characterization of (A) DSPE-PEG(2000)-(KKEEE)_3_K (expected m/z: 5171 g/mol), (B) DSPE-PEG(2000)-Met (expected m/z 2895 g/mol), and (C) DSPE-PEG(2000)-Cy7 (expected m/z: 3522 g/mol).

Figure S2. Phosphorylated AMPK to total AMPK level obtained *via* ELISA using 3D cysts (**p* ≤ 0.05, ***p* ≤ 0.01).


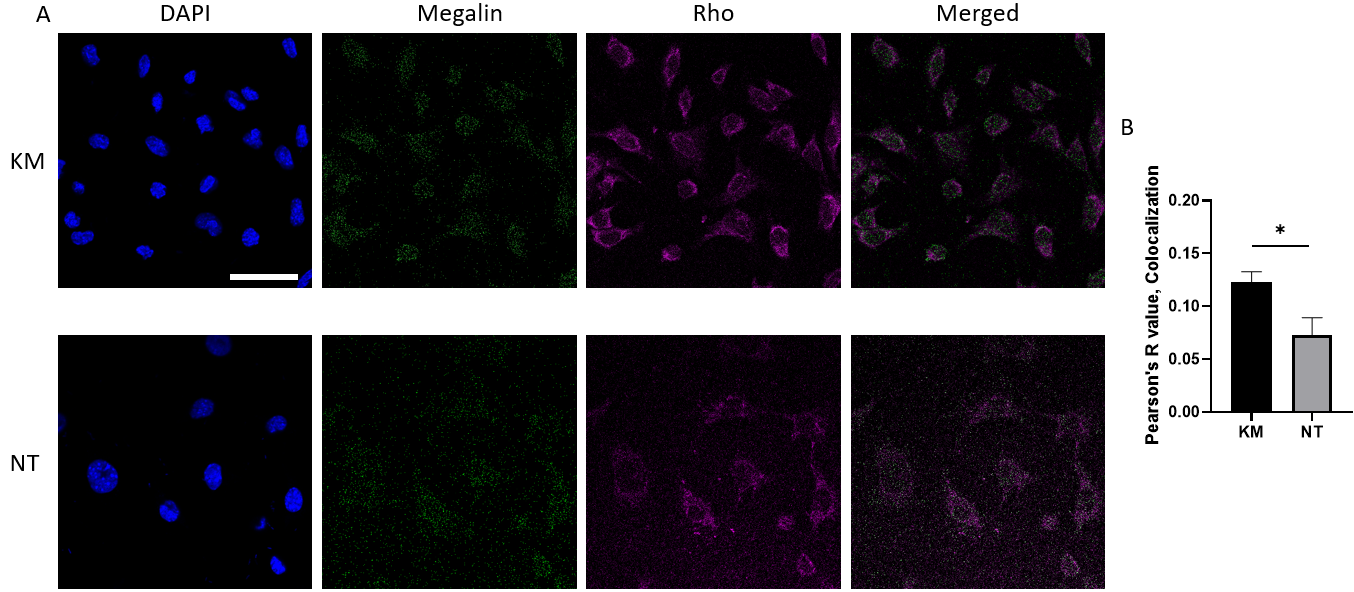


Figure S3. Megalin staining and colocalization with (A) KM and NT after incubation for 15 mins. (B) Quantification of colocalization between Rho and megalin. Scale bar: 100 μm. (**p* ≤ 0.05)


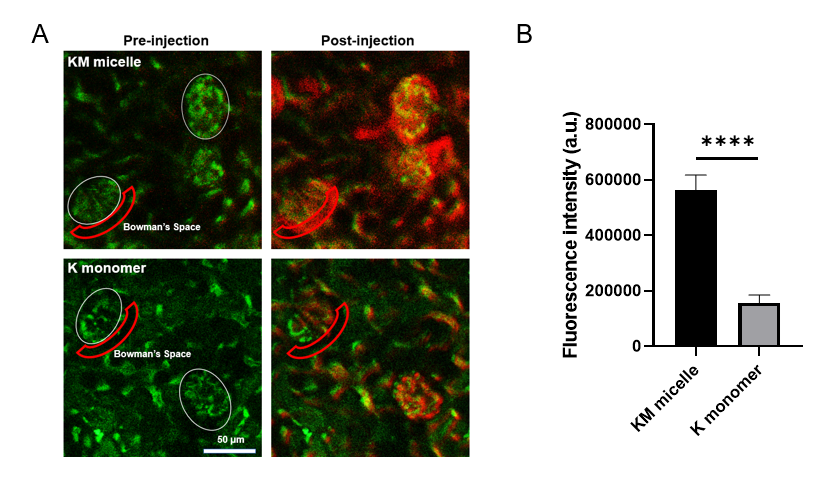


Figure S4. (A) Intravital images of kidney glomeruli (white circle) after KM and kidney-targeting amphiphiles (Cy7-DSPE-PEG2000-(KKEEE)_3_) were injected into the carotid artery of C57BL/6 mice. Both KM and kidney-targeting amphiphiles entered the glomerulus, but only KM micelles were able to pass through the GFB and enter the Bowman’s space. (B) Quantitative analysis showed significantly higher fluorescence intensity of 500kDa Dextran-Alexa Fluor 488 (green, plasma), Cy7-labeled KMs or amphiphiles (red, *****p* ≤ 0.0001).


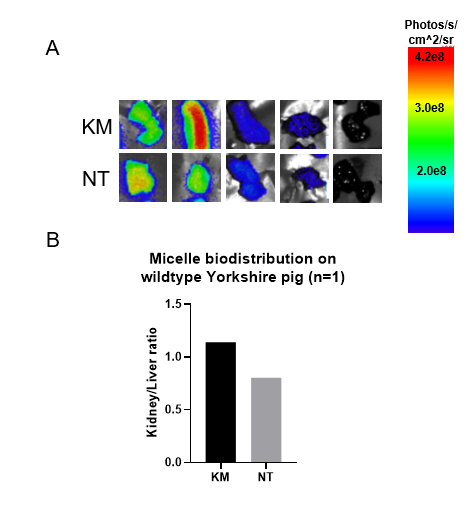


Figure S5. (A) Biodistribution of Cy7-labeled KM and NT micelles 24 hours after IV administration into Yorkshire pig via an ear vein catheter. (B) Quantification of *ex vivo* organ Cy7 fluorescence levels (n=1)


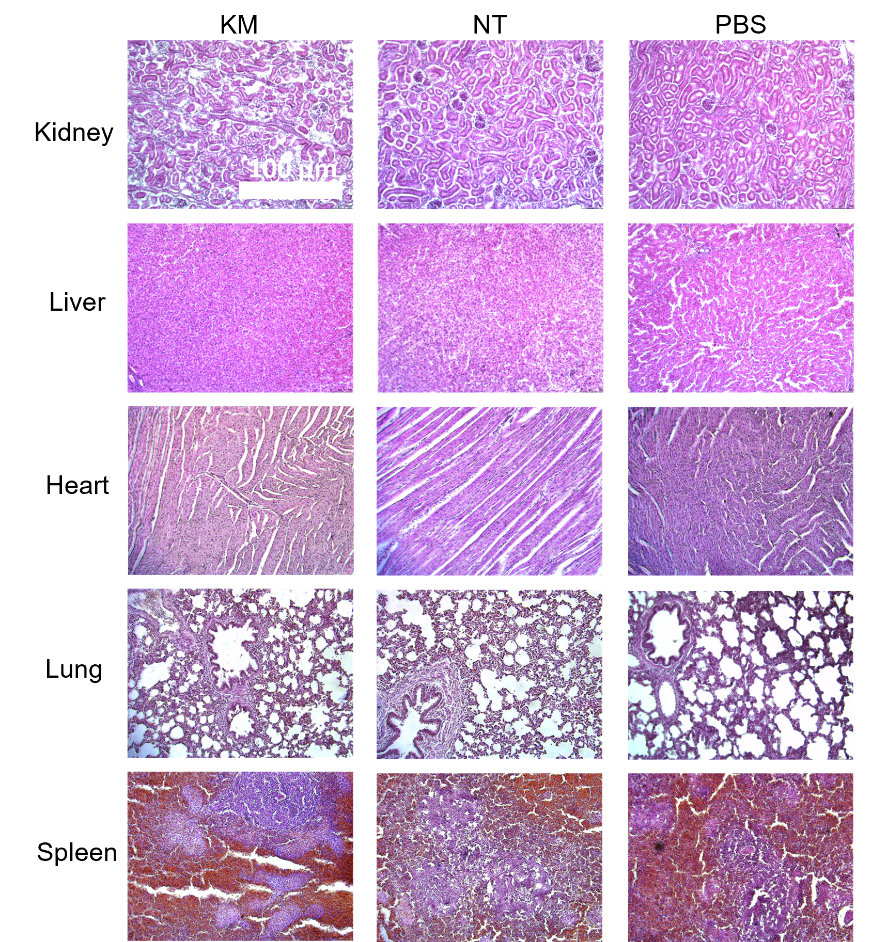


Figure S6. H&E staining of pig organ sections 24 hr after KM, NT or PBS injection.


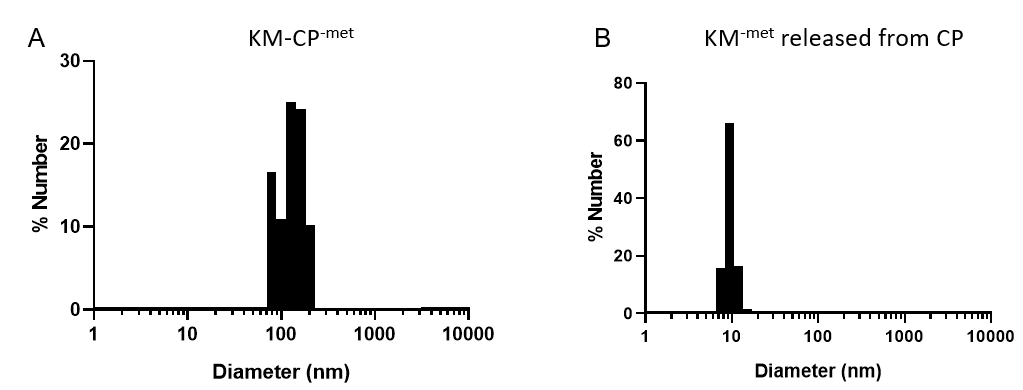


Figure S7. Representative DLS measurements of KM-CP^-met^ and KM^-met^ released from CP.


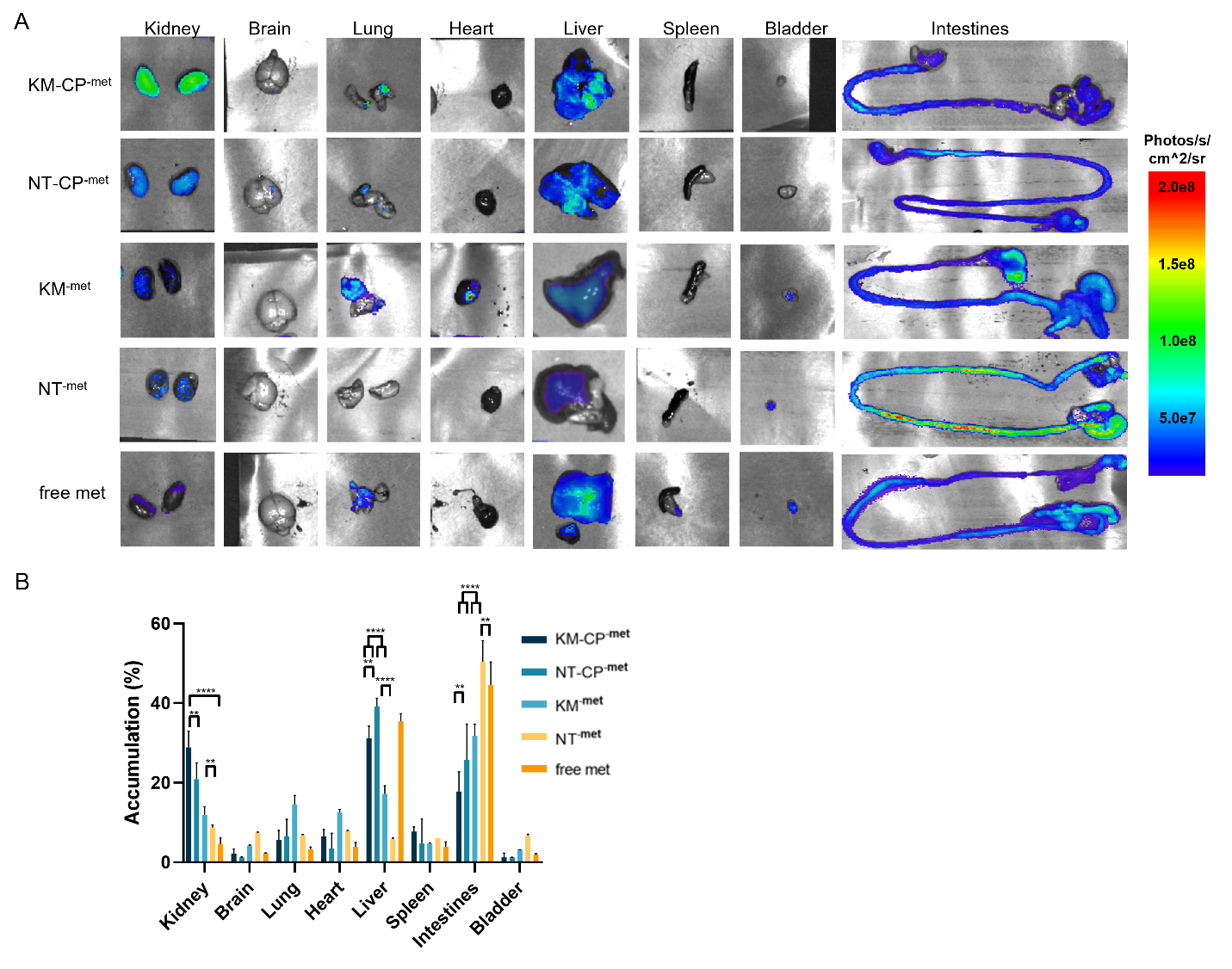


Figure S8. (A) Biodistribution of Cy7-labeled KM-CP^-met^, NT-CP^-met^, KM^-met^, NT^-met^, and free metformin 24 hours after oral gavage. (B) Quantification of *ex vivo* organ Cy7 fluorescence levels (**p* ≤ 0.05, ***p* ≤ 0.01, *****p* ≤ 0.0001).


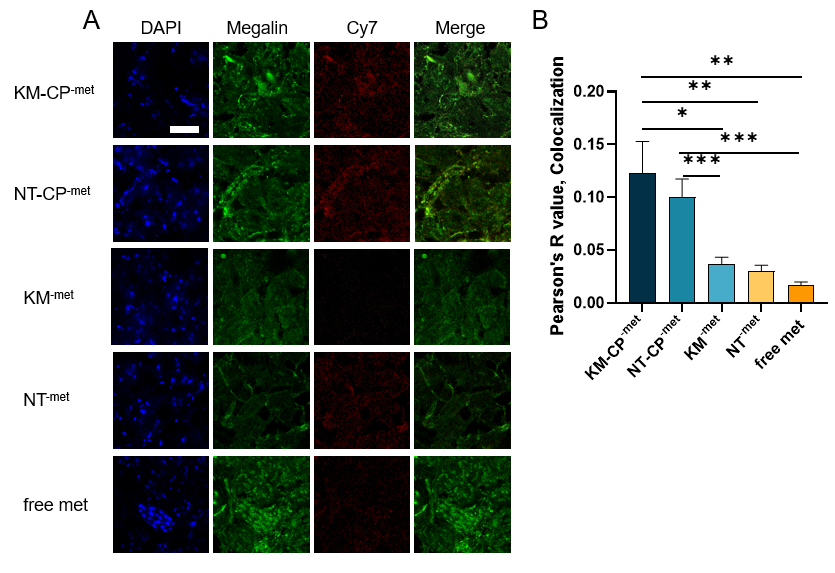


Figure S9. Megalin staining and colocalization with (A) KM-CP^-met^, NT-CP^-met^, KM^-met^, NT^-met^, and free metformin 24 hours after oral gavage. (B) Quantification of colocalization between Cy7 and megalin. Scale bar: 100 μm. (**p* ≤ 0.05, ***p* ≤ 0.01)


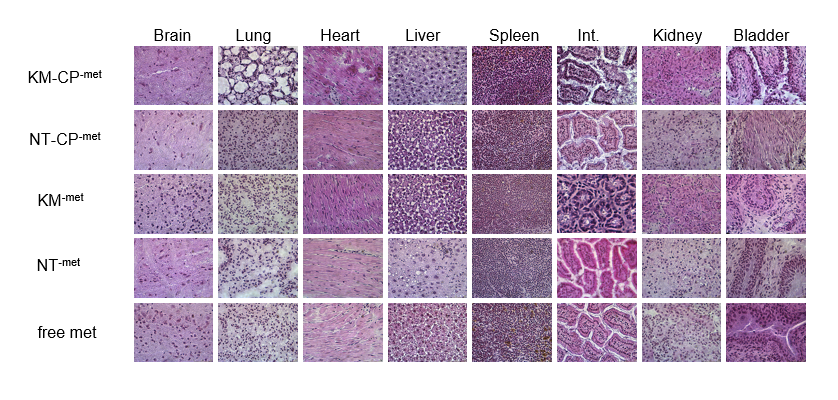


Figure S10. H&E staining of organ sections 24 hr after KM-CP^-met^ oral gavage.

Video S1. Intravital imaging of KM injected into the canulated carotid artery of C57BL/6 mice.

Video S2. Intravital imaging of kidney-targeting amphiphiles injected into the canulated carotid artery of C57BL/6 mice.
